# Serum Level of Protein-Bound Uraemic Toxins in Haemodialysis Patients with Chronic Kidney Disease-Associated Pruritus: Myths and Facts

**DOI:** 10.3390/jcm12062310

**Published:** 2023-03-16

**Authors:** Karolina Świerczyńska-Mróz, Danuta Nowicka-Suszko, Mariusz G. Fleszar, Paulina Fortuna, Piotr K. Krajewski, Magdalena Krajewska, Rafał Białynicki-Birula, Jacek C. Szepietowski

**Affiliations:** 1 Department of Dermatology, Venereology and Allergology, Wroclaw Medical University, 50-368 Wroclaw, Poland; 2 Department of Medical Biochemistry, Wroclaw Medical University, 50-368 Wroclaw, Poland; 3 Department of Nephrology and Transplantation Medicine, Wroclaw Medical University, 50-556 Wroclaw, Poland

**Keywords:** protein-bound uraemic toxins, indoxyl sulfate, p-Cresol sulfate, chronic-kidney-disease-associated pruritus, haemodialysis patients

## Abstract

Recent studies place great importance on Protein-Bound Uraemic Toxins (PBUT) in the context of etiopathogenesis of chronic kidney disease-associated pruritus (CKD-aP). This study aimed to investigate the possible contribution of free and total Indoxyl Sulfate (IS) and p-Cresol Sulfate (PCS) to the cause of CKD-aP. Group A included 64 patients on maintenance haemodialysis (HD) with CKD-aP. Group B included 62 patients on maintenance HD that did not report CKD-aP, and group C included 50 healthy controls. Pruritus severity was assessed using a Numerical Rating Scale (NRS). Moreover, other tools like UP-Dial, ItchyQoL, and the 4-Item Itch Questionnaire evaluating CKD-aP were completed by the patients. The serum levels of free and total IS and PCS concentrations were measured using the Ultra Performance Liquid Chromatography System. No significant difference in the serum level of free and total IS, or PCS, was observed between the patients who reported CKD-aP and those without pruritus. Moreover, there was no correlation between serum IS or PCS levels and the severity of the itch. Our study does not support earlier findings about higher levels of IS and PCS in patients reporting CKD-aP. Further studies will be needed to investigate these discrepancies as well as to understand the cause of CKD-aP.

## 1. Introduction

Chronic itch (CI) is defined as itching lasting 6 weeks or more. It is commonly associated with many dermatological and systemic diseases, such as diabetes mellitus, hypothyroidism, chronic hepatobiliary conditions, malignancies, and end-stage renal disease (ESRD) [1,2]. The term “uraemic pruritus” is widely used in the literature. However, in accordance with the latest research and a lack of clear dependence between uraemia and itch sensation, world experts in this field proposed “chronic kidney disease-associated pruritus” (CKD-aP) as a more precise nomenclature [3]. CKD-aP affects approximately 40% of patients undergoing maintenance haemodialysis (HD) [4]. It is considered a burdensome, frequent dermatological symptom in patients with ESRD and negatively impacts patients’ quality of life [5]. Researchers around the world have proven that this condition has a negative influence on sleep and daily activities, aggravates the symptoms of depression, and increases the risks of mortality and hospitalization in HD patients [6,7]. Surprisingly, in relation to the importance of the problem, there is a noticeable underestimation of CKD-aP in clinical practice. The reason for this may be the lack of knowledge on effective therapies leading to the neglect of this issue [8,9]. The effective treatment of CKD-aP is very often intractable and remains a challenge for dermatologists and nephrologists. This is due to this recurring and complex condition’s multifactorial and not fully understood causes [10]. However, an increasing number of ongoing studies and discoveries in the context of pruritus etiopathogenesis may bring revolutionary therapeutic possibilities. The most common theories explaining the development of CKD-aP include immune dysregulation, neuronal dysregulation, xerosis, disbalance in calcium and phosphorous metabolism, hyperparathyroidism, altered opioid transmission, and uraemic toxins (UTs) [11]. UTs are substances that are retained in the organism by patients with advanced kidney failure and can negatively interact with the biological functions of the organism [12]. In 2021, the European Uremic Toxin (EUTox) database listed 130 substances [13]. UTs can be divided into three groups: free water-soluble low molecular weight molecules (LMWM) (<0.5 kDa), middle molecules (0.5–60 kDa), and protein-bound UTs (PBUT). The latter mentioned have been in the spotlight lately in the context of interfering with the biochemical functions of individuals suffering from advanced stages of chronic kidney disease [14]. PBUT are molecules that circulate in the blood in free solute form and protein bound form and are barely removed during the process of HD [15,16]. Certain compounds belonging to PBUT, like indoxyl sulfate (IS) and p-Cresol sulfate (pCS), have been widely investigated regarding chronic kidney disease and its complications. So far, published studies emphasize the key role of the intestinal microbiome in the generation of UTs [17]. Both p-Cresol sulfate and indoxyl sulfate arise from bacterial protein fermentation in the large intestine [15]. However, their chemical structures differ—indoxyl sulfate belongs to the indole group, and p-Cresol sulfate belongs to phenols [12]. Most of these two substances circulate noncovalently bound to albumin and compete for the same albumin-binding sites (Sudlow site II) [15]. The study estimated the correlation between PBUT concentrations and renal dysfunction and revealed that both indoxyl sulfate and p-Cresol sulfate levels increased when renal function declined [18]. Moreover, the role of these compounds in the context of CKD complications has been extensively estimated in the last decade. IS may play a significant role in vascular diseases and higher mortality in CKD patients [19]. Similarly, free serum levels of p-Cresol are associated with worsened outcomes and may be considered a novel cardiovascular risk factor in haemodialysis patients [20,21]. The data on the potential contribution of PBUT in CKD-aP is very limited; however, this theory has recently been notoriously raised by experts in the field. The research conducted on 320 CKD patients proved not only significantly higher serum levels of total IS (*p* = 0.008) and PCS (*p* < 0.0001) among participants reporting CKD-aP but also a correlation between itch severity and total PCS concentration (*p* = 0.002) [22]. Nonetheless, other investigators did not confirm these findings, and showed no significant association between the 5D score and PBUTs serum concentrations [23]. Another interesting hypothesis assumes that UTs may affect protease-activated receptor-2 (PAR-2) expression in the skin of CKD subjects and thus may lead to the development of CKD-aP [24]. These studies are discussed in more detail in the discussion paragraph. We decided to perform this detailed study to verify earlier ambiguous and limited findings on this topic. Therefore, the aim was to investigate the possible contribution of free and total indoxyl sulfate and p-Cresol sulfate to the pathogenesis of itch in patients undergoing maintenance haemodialysis.

## 2. Materials and Methods

### 2.1. Study Population

This study enrolled 174 adult subjects. The study groups were formed depending on itch sensations reported by the CKD patients. Group A included 61 patients on maintenance HD with CKD-aP. Group B included 63 patients on maintenance HD that did not report CKD-aP, and group C included 50 healthy controls. The control group corresponded in age and gender to the patients from the study group and had no history of any pruritic or systemic diseases. The study group included patients over 18 years of age receiving haemodialysis 3 times a week for at least 3 months, who signed the patient’s informed consent. The following exclusion criteria were respected: primary disorders causing itch, psychiatric disorders or communication problems, antipruritic therapy, and lack of informed consent. All participants underwent a physical and dermatological examination. Basic demographic and medical data, including the cause of renal failure, duration of HD, type of vascular access, and previous treatment of pruritus, were collected. This research obtained ethical approval from the Wroclaw Medical University Ethics Committee (Consent no. 26/202, date: 29 January 2021). All patients provided their written informed consent to participate in this study.

### 2.2. Laboratory Tests

Quantitative analysis of Indoxyl Sulfate and p-Cresol Sulfates was carried out on an LC-QTOF-MS system consisting of an Acquity Ultra-Performance Liquid Chromatography System (Waters, Milford, MA, USA) and quadrupole time-of-flight mass spectrometer (Xevo G2 Q-TOF MS, Waters, Milford, MA, USA). At inclusion to the study, blood samples of 9 mL were taken immediately before the dialysis session from all the participants.

#### 2.2.1. Chemicals

Indoxyl sulfate potassium salt, Indoxyl sulfate potassium salt-d5, and p-Cresol sulfate potassium salt were procured from Cayman Chemicals (Ann Arbor, MI, USA). p-Cresol sulfate potassium salt-d7 was obtained from Cambridge Isotope Laboratories (Tewksbury, MA, USA). Methanol, acetonitrile (ACN), water, ammonium formate, and formic acid (FA) were acquired from Merck Millipore (Warsaw, Poland).

#### 2.2.2. Sample and Calibration Standards Preparation and LC-MS Analysis

Indoxyl Sulfate and p-Cresol Sulfate sample quantitative UHPLC-ESI-QTOF-MS analysis. Samples and calibration standards for quantitative analysis of Indoxyl Sulfate and p-Cresol sulfate were procured in the same manner. Briefly, 100 µL of calibration standards or serum samples were placed into 2.0 mL polypropylene tubes with 10 µL of internal standard solution in methanol (40 µg/mL of Indoxyl Sulfate-d5 and p-Cresol sulfate-d7) then after a minute of mixing samples were deproteinized with 400 µL of acetonitrile at 25 °C for 10 min and centrifuged at 12,000 RCF for 7 min at 4 °C. The 100 µL of obtained supernatant was diluted with 400 µL of water and transferred into autosampler glass vials. For free non-bind with protein–forms, 200 µL of calibration standards or serum samples were placed into 2.0 mL polypropylene tubes with 20 µL of internal standard solution in methanol (40 µg/mL of Indoxyl Sulfate-d5 and p-Cresol sulfate-d7) then after a minute of mixing samples were transferred and centrifuged for 50 min at 10,000× *g* at 20 ℃ with a 3000 MWCO filter (Merck Millipore, Burlington, MA, USA). Subsequently, samples were prepared in the same way as the total form. Quantitative analysis of Indoxyl Sulfate and p-Cresol Sulfates was carried out on an LC-QTOF-MS system consisting of an Acquity UPLC system (Waters, Milford, MA, USA) and quadrupole time-of-flight mass spectrometer (Xevo G2 Q-TOF MS, Waters, Milford, MA, USA). Analytes were separated using Waters HSS T3 chromatographic column (1.0 × 50 mm, 1.75 µm) with a linear gradient from 3% to 95% of mobile phase B in 9.5 min with a total flow rate of 180 µL/min. The mobile phases of 0.1% FA in water (A) and 0.1% FA in acetonitrile (B) were used. Data acquisition was carried out with an electrospray ionization (ESI) ion source operated in negative mode. Source parameters were as follows: nebulizing and drying gas (nitrogen): 700 L/h and 30 L/h, respectively; spray voltage: 2.5 kV; source temperature: 120 °C; and desolvation temperature: 350 °C. The scan range was 105–800  m/z for all acquisition events. The target metabolites were quantified based on their extracted ion chromatograms (EIC)—212.0018 m/z, 187.0065 m/z, 217.0331 m/z, and 194.0504 m/z for Indoxyl Sulfate, p-Cresol sulfate, Indoxyl Sulfate-d5, and p-Cresol sulfate-d7, respectively.

### 2.3. Pruritus Assessment

The evaluation of pruritus was carried out multidimensionally by using well-known tools as well as some newly validated questionnaires. Itch intensity was assessed using the NRS, a widely used and preferred instrument to evaluate the intensity of pruritus. Patients indicated, on the eleven-point scale, the worst itch intensity they perceived during the last 3 days. The severity of pruritus was classified as follows: NRS < 3—mild pruritus, NRS ≥ 3 and <7—moderate pruritus, NRS ≥ 7 and <9—severe pruritus, and finally, NRS ≥ 9—very severe pruritus [25]. Another tool used in the study was 4IIQ, which consists of 4 questions concerning the distribution, intensity, and frequency of the itch and sleep disturbances reported by the patients. The possible score ranges from 3 to 19 points [26]. Moreover, a Polish, recently validated version of the UP-Dial questionnaire was employed. UP-Dial characterizes itch, especially in dialysis patients, and consists of 14 multidimensional questions. Three domains of this questionnaire precisely analyze all aspects of chronic itch: frequency, intensity, distribution, skin lesions caused by itch, sleep disorders, and the psychosocial status of the patient [27]. Finally, to evaluate the influence of chronic itch on QoL, a Polish version of the ItchyQoL questionnaire was filled out. It is a validated 22-question instrument with three dimensions: symptoms, functional limitations, and emotions. Questions are scored on a 5-point scale, and the total score ranging between 22 and 110 points estimates QoL [28].

### 2.4. Statistical Analysis

The IBM SPSS Statistics v. 26 software (SPSS Inc., Chicago, IL, USA) was used for the statistical analysis. All data were checked for normal or abnormal distribution. For quantitative data analysis, the Mann–Whitney U test and Spearman’s correlations were used. Assessment of qualitative results was evaluated with the use of the chi-squared test. Data were expressed as mean ± SD, median, first and third quantiles, with *p* < 0.05 considered statistically significant.

## 3. Results

### 3.1. Baseline Characteristics of the Subjects

In this cross-sectional study, 174 subjects were included. Females constituted 47% (n = 82) of the study population and males 53% (n = 92). The mean age of the participants was 57.46 ± 16.24 years. All HD patients were undergoing haemodialysis using high-flux polysulfone membrane dialyzers. The average dialysis vintage was 49.58 ± 51.9 months, and the haemodialysis was performed using bicarbonate dialysate. The single pool Kt/V accessing the dialysis adequacy for the whole study population was 1.25 (SD ± 0.40). In a group of patients reporting CKD-aP, it was 1.13 (SD ± 0.32); in a group of patients not reporting CKD-aP, it was 1.34 (SD ± 0.44). We noted no statistically significant difference between these two groups. Thus, the Kt/V level did not play any important role in perceiving pruritus in our study group. Only 35% of HD patients underwent dialysis through a tunneled internal jugular central venous catheter and the rest through an artery-venous fistula. The dominant causes of renal failure in the study group were glomerulonephritis (n = 24, 19.4%) and diabetic nephropathy (n = 24, 19.4%). Moreover, statistical analysis of individual groups showed no significant differences in terms of gender, age, dialysis vintage, and cause of renal failure.

### 3.2. Serum Levels of Free and Total Indoxyl Sulfate and p-Cresol Sulfate

The results showed that free and total IS and PCS serum levels were significantly higher both in HD patients with pruritus and HD patients without pruritus compared to the healthy controls (*p* < 0.001). However, no significant difference in the serum level of free and total IS, or PCS, was observed between the patients who reported CKD-aP and those without pruritus. The comparison of free and total Indoxyl Sulfate and p-Cresol Sulfate serum level in three study groups is presented in Table 1.

### 3.3. Pruritus Assessment and Serum Levels of PBUT

The mean intensity of pruritus assessed by the Numerical Rating Scale (NRS) in patients undergoing maintenance haemodialysis was 4.87 ± 2.21 points. According to the NRS cut-offs, moderate pruritus was reported most frequently—in 60.65% of the cases. Mild pruritus was experienced by 14.75%, severe by 21.31%, and very severe only by 3.27% of the study population. The mean Uraemic Pruritus in Dialysis Patients (UP-Dial) total score in the itchy group was 14.31 ± 9.85 points. Additionally, the mean score of the 4-Item Itch Questionnaire (4IIQ) and ItchyQoL in this group was assessed as 8.44 ± 3.64 points and 36.84 ± 13.65 points, respectively. More detailed data are presented in Table 2.

However, no correlation was found between the serum level of PBUT and the severity of itch assessed by NRS. Likewise, the total scores of other instruments used in this study (Up-Dial, 4IIQ, and ItchyQoL) also did not reveal significant relationships with serum concentrations of studied PBUT (Table 3).

## 4. Discussion

In recent decades, the negative impact of PBUT on the proper functioning of the organism and the relationship between their serum level and patients’ clinical outcomes were highlighted in a large number of studies [18,19,20,21,29,30,31]. Due to their strong tendency to bind with albumins, the process of elimination during standard haemodialysis is very restrained. Toxin accumulation may lead to various side effects, including higher mortality risk, cardiovascular complications, and infections [18]. IS has proved to have a multifactorial nephrotoxic impact. Firstly, by enhancing the expression of transforming growth factor-β1 (TGF-β1), tissue inhibitor of metalloproteinase-1 (TIMP-1) and pro-α-1(I)-collagen and stimulating both tubulointerstitial fibrosis and glomerular sclerosis [32]. Moreover, it may induce renal hypoxia by stimulating oxidative stress, which is followed by endothelial senescence [33]. Additionally, one cannot fail to mention the pro-inflammatory effects of IS [34]. The toxicity of PCS also comes from stimulating the generation of reactive oxygen species and intensifying cytotoxic effects on renal tubular cells [14]. Similarly to IS, PCS increases the production of inflammatory cytokines (TGF-β1, TIMP-1, pro-α-1(I)-collagen), causing renal fibrosis [35]. What is more interesting, Lin et al. suggested p-Cresol sulfate levels as a valuable marker in the prediction of cardiovascular disease and kidney function deterioration in CKD patients without dialysis (stage 3–5) [36]. Analysis showed that a serum level of p-Cresol sulfate > 6 mg/L was significantly related to these complications during a 3-year follow-up [36]. In summation, the conducted studies definitely proved that the influence of IS and PCS on the course of CKD is indisputable. So far, published studies have emphasized that systemic inflammation may contribute to the pathogenesis of CKD-aP. Therefore, the possible proinflammatory role of PBUT may play a role in its pathogenesis. On the other hand, the impact of these toxins on chronic pruritus may be doubtful; only a few research groups have explored this issue so far, with ambiguous results.

Proteinase-activated receptor-2 (PAR-2) was first associated with a novel non-histaminergic pruritic pathway in research performed on patients suffering from atopic dermatitis (AD). Steinhoff et al. revealed not only an enhanced level of this receptor in skin biopsies of AD patients but also proved itch sensations appearing after intracutaneous injection of endogenous PAR-2 agonist [37]. Subsequently, a pilot study conducted on 12 ESRD patients with pruritus, four ESRD patients without pruritus, and six healthy controls documented higher serine protease activity in skin samples taken from pruritic patients. Moreover, a positive correlation was observed between PAR-2 expression and itch intensity assessed with Visual Analogue Scale (VAS) [38]. Finally, Kim et al. performed a detailed study investigating the effect of PBUT on the PAR-2 expression in the skin [24]. Results showed that indoxyl sulfate, p-cresol, and sera from CKD patients significantly induced PAR-2 mRNA and protein expression in the cultures of normal human epidermal keratinocytes (NHEK). Moreover, skin samples from patients with CKD and from mice with CKD presented increased PAR-2 expression compared to healthy controls. These findings support the potential contribution of uraemic solutes in the pathogenesis of CKD-aP. Another study associating PBUT and CKD-aP, performed on 320 CKD (stage 1–5) participants, reported significantly higher serum concentrations of total IS (*p* = 0.008) and PCS (*p* < 0.0001) among patients with pruritus symptoms [22]. After further adjustments with anthropometric variables, fasting glucose, total cholesterol, glutamic pyruvic transaminase, uric acid, albumin, WBC count, and hs-CRP, the PCS concentration was still significantly associated with pruritus. On the contrary to IS serum levels, in this case, the relationship between pruritus disappeared after adjustments for glutamic pyruvic transaminase and uric acid concentrations. Moreover, Wang et al. also found a correlation between total PCS level and itch severity (*p* = 0.0002) [22]. Authors emphasized the possible proinflammatory role of PCS, which through systemic inflammation, could contribute to the pathogenesis of CKD-aP. Unfortunately, Yamamoto et al. did not support these results and showed no significant associations between the 5D score and serum concentrations of the various PBUTs (IS, PCS, phenyl sulfate, and hippuric acid) [23].

Our study thoroughly estimated total and free concentrations of PBUT in patients undergoing haemodialysis. A major part of both IS and PCS are bound with albumins, which hinders their clearance. Only concentrations of free PBUT molecules may differ in terms of dialysis. Therefore, it is reasonable to assume that serum-free toxin levels may correlate with pruritus sensation or its severity. Unluckily, our results did not confirm this theory. Both free and total serum levels of IS and PCS were not statistically different between individuals reporting CKD-aP and those not reporting this symptom. The difference was observed only between healthy controls and patients undergoing HD (*p* < 0.001), which is clearly expected due to the accumulation of UTs in patients with ESRD. Otherwise, no correlation between concentrations of PBUT and itch severity was observed. In this study, a reliable and widely used tool—NRS, was employed to estimate the severity of itch, but also three other questionnaires which precisely characterized pruritus and described its influence on the quality of life (QoL) were in use. Despite prior reports, none of the total scores achieved by our participants correlate with serum levels of IS or PCS. These discrepancies between our research and previous studies may appear due to differences between studied populations, coexisting comorbidities, or even differentiated contributions of individual potential etiopathogenetic factors of CKD-aP among HD patients.

In case of confirmation of previous discoveries in this issue, it would be reasonable to use PBUT as a target for new antipruritic therapies. One past study showed a decrease in serum indoxyl sulfate concentration after administration of oral AST-120, which absorbs the indole—the precursor of indoxyl sulfate in the intestine, in a group of HD patients. Furthermore, the diminution of IS serum levels went hand in hand with the alleviating of CKD-aP [39]. Reduction in itch was observed in 9 of 10 patients, and as many as five patients reported complete remission of general pruritus. However, the initial IS serum level in a group of CKD-aP patients did not statistically differ from HD patients not reporting itch; 12 weeks after administration of AST-120, it declined significantly (*p* < 0.05) [39]. To the best of our knowledge, there is a lack of other recent studies reevaluating the mechanism of reducing CKD-aP. Moreover, AST-120 was recognized many times as effective in inhibiting the progression of kidney failure and has been accepted in most Asian countries as a therapeutic strategy for CKD [40]. Its influence on the elimination of IS decreases oxidative stress in tubular cells, mesangial cells, vascular smooth muscle cells, endothelial cells, and osteoblasts. Thus, it contributes to slowing down the cardiovascular disease and osteodystrophy of CKD patients [41]. Notwithstanding, another two randomized controlled trials did not support the beneficial aspect of long-term use of AST-120 in patients with advanced renal dysfunction [42,43]. On the other hand, as the proper method of removing PBUT remains uncertain, and their formation is stimulated by bacteria, recently gut microbiota has been considered a promising target to decrease toxins levels [44].

Despite suggestive results in the context of PBUT and findings presented in our study, the general role of UTs in the etiopathogenesis of CKD-aP should not be depreciated. Several middle molecules, which are included as uraemic toxins, were linked to CKD-aP. A higher concentration of β_2_–Microglobulin (β_2_-M) was associated with severe CKD-aP in a study conducted on 1773 participants. Additionally, lower β_2_-M levels played a protective role in patients’ outcomes, contrary to severe itch, which increased mortality risk [45]. Authors of this research assumed that increased concentrations of β_2_-M might stimulate the production of cytokines—IL-2 or TNF-α, which activate CD4 lymphocytes and raise the risk of CKD-aP. Kimmel et al. revealed the dependency between pruritus reporting by 171 HD patients and IL-6 serum level, thereby emphasizing the central role of microinflammation in the etiopathogenesis of this condition [46]. In addition, some free water-soluble low molecular weight molecules like uric acid were repeatedly evaluated in the context of CKD-aP, however, with different results [22,47]. Undoubtedly, UTs and other potential components of the pathogenesis of pruritus may serve as a target for new antipruritic therapies. Despite numerous studies on this topic, there are still no clear guidelines for the management of patients with CKD-aP and no objective comparison of available pharmacological methods [48]. This situation leads to different choices among doctors in clinical practice, and effects are often unsatisfactory and difficult to achieve. Nevertheless, it should be emphasized that difelikefalin is the first drug approved in August 2021 by the Food and Drug Administration (FDA) and in April 2022 by the European Medicines Agency (EMA) for the treatment of moderate-to-severe pruritus associated with CKD in adults undergoing haemodialysis [49]. Due to its efficacy and acceptable safety profile, it should be currently regarded as the primary treatment in a group of HD patients with refractory itch sensation [49,50].

The limitation of this cross-sectional study is restricted to dialysis stations only in two settings. The evaluation of the molecules level was performed in a one-point measurement. Moreover, the study assessed only PBUT without other inflammation markers and was narrowed to only HD patients, excluding CKD patients or individuals on peritoneal dialysis. Due to the unobvious role of toxins in CKD-aP, bigger multicenter studies, with an evaluation of additional, other proinflammatory markers, can bring even more valuable results.

## 5. Conclusions

In summary, so far, published research regarding the role of PBUT in CKD-aP etiopathogenesis is ambiguous and very limited. Our study does not support earlier findings about higher levels of IS and PCS in patients reporting CKD-aP. Further studies will be needed to investigate these discrepancies as well as to understand the cause of itch in CKD patients.

## Figures and Tables

**Table 1:** Concentrations of free and total Indoxyl Sulfate and p-Cresol Sulfate in HD patients reporting pruritus (group A), not reporting pruritus (group B), and healthy controls (group C).

Parameter (μg/mL)	All HD Patients (n = 124)	Group A—Reporting Itch (n = 61)	Group B—Not Reporting Itch (n = 63)	Group C—Healthy Controls (n = 50)	*p*-Value Group A vs. Group B	*p*-Value Group A vs. Group C	*p*-Value Group B vs. Group C
	Median (Q1; Q3)						
**Indoxyl sulfate total**	24.1 (11.7; 33.8)	26.7 (12.3; 36.3)	23.8 (10.3; 33.2)	0.9 (0.5; 1.1)	NS	*p* < 0.001	*p* < 0.001
**Indoxyl sulfate free**	2.6 (1.3; 3.9)	2.8 (1.4; 4.1)	2.4 (1.1; 3.8)	0.1 (0.06; 0.2)	NS	*p* < 0.001	*p* < 0.001
**Cresol sulfate total**	28.6 (15.1; 38.9)	28.5 (14.0; 40.3)	28.7 (16.5; 36.9)	1.9 (1.0; 3.2)	NS	*p* < 0.001	*p* < 0.001
**Cresol sulfate free**	3.2 (1.5; 4.9)	3.1 (1.6; 5.4)	3.3 (1.7; 4.7)	0.2 (0.1; 0.3)	NS	*p* < 0.001	*p* < 0.001

NS—not statistically significant, SD—standard deviation.

**Table 2:** Pruritus Assessment in hemodialysis patients.

Tool	Mean, SD	Minimum Score (Points)	Maximum Score (Points)	Amount of Patients according to Itch Severity *
NRS	4.87 ± 2.21	1	10	mild: n = 9 moderate: n = 37 severe: n = 13 very severe: n = 2
ItchyQoL	36.84 ± 13.65	22	80	
4IIQ	8.44 ± 3.64	4	18	
UP-Dial	14.31 ± 9.85	2	54	

NRS—Numerical Rating Scale, 4IIQ—4-Item Itch Questionnaire, Up-Dial—Uraemic Pruritus in Dialysis Patients, * Severity of pruritus was classified as follows: NRS < 3—Mild pruritus, NRS ≥ 3 and <7—Moderate pruritus, NRS ≥ 7 and <9—Severe pruritus, finally NRS ≥ 9—Very severe pruritus.

**Table 3:** Relationship between the serum level of PBUT and total scores of itch questionnaires.

Parameter	NRS	ItchyQoL	4IIQ	UP-Dial
Indoxyl sulfate total	r = −0.0148 *p* = 0.254	r = −0.081 *p* = 0.535	r = 0.005 *p* = 0.97	r = −0.128 *p* = 0.324
Indoxyl sulfate free	r = −0.25 *p* = 0.846	r = −0.005 *p* = 0.969	r = 0.096 *p* = 0.464	r = −0.011 *p* = 0.932
Cresol sulfate total	r = 0.18 *p* = 0.164	r = 0.125 *p* = 0.336	r = 0.098 *p* = 0.454	r = 0.057 *p* = 664
Cresol sulfate free	r = 0.213 *p* = 0.099	r = 0.212 *p* = 0.1	r = 0.201 *p* = 0.121	r = 0.183 *p* = 0.159

NRS—Numerical Rating Scale, 4IIQ—4-Item Itch Questionnaire, Up-Dial—Uraemic Pruritus in Dialysis Patients, *p*—*p*-Value, r—Correlation coefficient.

## Data Availability

The datasets generated and analyzed in the current study are available from the corresponding author on reasonable request.
